# Cancer incidence in children and young adults did not increase relative to parental exposure to atomic bombs

**DOI:** 10.1038/sj.bjc.6601322

**Published:** 2003-10-28

**Authors:** S Izumi, K Koyama, M Soda, A Suyama

**Affiliations:** 1 Department of Statistics, Radiation Effects Research Foundation (RERF), 5-2 Hijiyama Park, Minami-ku, Hiroshima 732-0815 Japan; 2 Department of Epidemiology, RERF, 5-2 Hijiyama Park, Minami-ku, Hiroshima, 732-0815 Japan; 3 Department of Epidemiology, RERF, 1-8-6 Nakagawa, Nagasaki, 850-0013 Japan

**Keywords:** cancer incidence, ionizing radiation, preconception exposure, cohort study

## Abstract

We have examined whether parental exposure to atomic bomb radiation has led to increased cancer risks among the offspring. We studied 40 487 subjects born from May 1946 through December 1984 who were cancer-free in January 1958. One or both parents were in Hiroshima or Nagasaki at the time of the bombing and for childbirth. Using population-based tumor registry data we analyzed cancer incidence data from 1958 to 1997 by Cox regression models, and we examined the effects of both paternal and maternal irradiation with adjustment for city, sex, birth year, and migration. During follow-up, 575 solid tumor cases and 68 hematopoietic tumor cases were diagnosed. Median age at diagnosis was 39.7 years. Median doses were 143 millisierverts for 15 992 exposed (5+ millisierverts or unknown dose) fathers and 133 millisierverts for 10 066 exposed mothers. Cancer incidence was no higher for subjects with exposed parents than for the reference subjects (0–4 millisierverts), nor did the incidence rates increase with increasing dose. For 3568 subjects with two exposed parents, the adjusted risk ratio for all cancer was 0.97 (95% confidence interval 0.70–1.36). Because of the small number of cases, however, we cannot exclude an increase in cancer incidence at this time.

Our long-term study of a large cohort of offspring of atomic-bomb survivors screens regularly for possible effects of parental preconception exposure to radiation. The latest mortality study (Izumi *et al*, 2003) suggested that such exposure did not lead to increased cancer mortality rates in childhood and young adulthood among the offspring born in Hiroshima and Nagasaki. This finding is consistent with earlier studies of the cohort, which found no dose-dependent increases in mortality (Kato *et al*, 1966; Neel *et al*, 1974; Yoshimoto *et al*, 1991; Little *et al*, 1994), childhood cancer (Yoshimoto *et al*, 1990), untoward pregnancy outcomes (Neel and Schull, 1956; Otake *et al*, 1990), cytogenetic abnormalities (Awa *et al*, 1987), or loss of enzyme activity (Neel *et al*, 1988). However, because cancers with high survival rates could not be adequately evaluated by mortality data, the effects of parental irradiation on cancer risks among the offspring need to be examined using the cancer incidence data.

In the present cohort study, we used population-based tumor registry data to examine whether parental exposure to atomic bomb radiation was associated with higher cancer incidence rates among the offspring born in Hiroshima and Nagasaki. We examined the effects of paternal and maternal irradiation on cancer risks among offspring both before and after they were 20 years of age.

## METHODS

### Study population

We analyzed a subset of the F_1_ mortality study cohort of the Radiation Effects Research Foundation (RERF), Japan (Kato and Schull, 1960; Yoshimoto *et al*, 1990), consisting of 40 487 Japanese offspring (20 743 men and 19 744 women) born from 1 May 1946 through 31 December 1984. They were conceived between 1 month and 38 years after the atomic bombing in August 1945 and one or both parents were in either city of Hiroshima or Nagasaki at the time of the bombing and for the childbirth. Almost two-thirds were born in Hiroshima, the majority between 1946 and 1959. Radiation dose was known for at least one parent. The subjects were alive and cancer-free in January 1958. Family and biological relationships were established from birth records, parental interviews, and maternal pregnancy data (Neel and Schull, 1956). The study sample did not include persons born to atomic-bomb survivors who had moved away from the cities after the bombings.

### Follow-up/identification of cancer cases

We assessed cancer incidence from 1 January 1958 through 31 December 1997 through population-based tumor registries in Hiroshima and Nagasaki. These registries have collected information from local hospitals and death certificates since 1957 for Hiroshima and 1958 for Nagasaki. Tissue registries have been providing supplemental information about tumor cases to the tumor registries. Neither a nation-wide tumor registry nor access to other tumor registries has been established. Details of the methodology have been published elsewhere (Mabuchi *et al*, 1994). The RERF Human Investigation Committee approved the research protocol for the F_1_ epidemiologic studies. Residency information for each cohort member was in-accessible because of privacy laws, so residence probabilities in the tumor registry catchment areas were used instead to adjust for the possible effects of migration on cancer risks (Sposto *et al*, 1992).

We classified the end points into two groups based on the *International Classification of Diseases*, 9th Revision (ICD-9) (World Health Organization, 1977): (1) hematopoietic tumors, including leukemia (ICD-9 204–208), Hodgkin's and non-Hodgkin's lymphoma (ICD-9 200–202), and multiple myeloma (ICD-9 203) and (2) other malignant (solid) tumors (ICD-9 140–199) together with benign brain and central nervous system tumors (ICD-9 225). We recorded final case status (cancer or cancer-free), age at which the individual ceased to contribute person-years of follow-up (age at diagnosis of first primary cancer, death, or the end of follow-up, whichever occurred first), and age at entry to the study.

### Parental data

Parental data included date of birth and gonadal dose as estimated by the DS86 dosimetry system (Roesch, 1987; National Academy of Science, 1990). Mean age at the time of bombing was 24.4 years (standard deviation 9.2) for fathers and 19.8 years (standard deviation 7.7) for mothers. As in recent mortality studies of atomic-bomb survivors (Pierce *et al*, 1996; Shimizu *et al*, 1999), we adjusted for dose error and used a weighted dose (the gamma dose plus 10 times the neutron dose). In contrast to certain previous studies of childhood cancer, this study examined paternal and maternal doses separately rather than combining them into a conjoint dose.

We divided parental doses into seven groups: 0–4 millisierverts (mSv) (reference group), 5–49, 50–149, 150–499, 500–4000, unknown dose (exposed, but with insufficient information on dose), and not in city at the time of the bombing. Doses above 4 mSv were divided into quartiles. We defined exposed parents as those with doses exceeding 4 mSv or with unknown dose. Dose measured in units of 100 mSv were used to test for a linear dose-response.

As shown in Table 1

**Table 1:** Distribution of parental radiation dose among 40 487 offspring of atomic-bomb survivors and controls

		**Paternal dose**							
**mSv (mean)**		**0–4^a^**	**5–49**	**50–149**	**150–499**	**500–4000**	**Unknown**	**Not in city**	**Total**
**Maternal dose**		**(0)**	**(22)**	**(91)**	**(290)**	**(1148)**	**(–)**	**(0)**	
0–4^a^	(0)	5322	758	511	570	430	524	3279	11 394
5–49	(22)	532	435	206	128	60	148	564	2073
50–149	(92)	476	174	285	90	45	111	667	1848
150–499	(291)	515	169	113	272	55	89	601	1814
500–4000	(1004)	628	121	58	105	158	103	739	1912
Unknown	(–)	931	216	136	169	122	0	845	2419
Not in city	(0)	9396	1597	1901	1974	1711	2448	0	19 027
Total		17 800	3470	3210	3308	2581	3423	6695	40 487

a Classified as the reference group.

, 22 490 (56%) had one or two exposed (5+ mSv or unknown dose) parents, while 3568 (8.8%) had two exposed parents. Median doses were 143 mSv for 15 992 exposed fathers (range 5–3835) and 133 mSv for 10 066 exposed mothers (range 5–3103). The association between paternal and maternal doses was low (correlation coefficient 0.02 for the subjects whose parents were both in city with known dose).

### Statistical methods

We analyzed cancer incidence rates for subjects both before and after they were 20 years of age. We used Cox regression models to compute risk ratios and 95% confidence intervals for paternal and maternal radiation dose (using either groups or continuous values) with adjustment for the baseline rates for city, sex, year of birth, age at entry, and residency. Residence probabilities were estimated by city (Hiroshima, Nagasaki), sex (male, female), and calendar period (1958–60, 61–65, 66–70, 71–75, 76–80, 81–85, 86–90, 91–97). We also checked proportionality of hazard rates (Therneau and Grambsh, 2000). We found in a preliminary analysis that the results from Poisson regression models were similar to those from Cox regression models. Heterogeneity of risk ratios was tested among the groups of subjects whose parents were in either city of Hiroshima or Nagasaki at the time of the bombing. Evidence of any linear dose-response was also investigated among the subjects whose parents were in either city at the time of the bombing with known dose. We calculated two-sided *P* values, with values less than 0.05 indicating significance. EPICURE software was used for all statistical analyses (Preston *et al*, 1993).

## RESULTS

During the 40-year period of follow-up (Table 2

**Table 2:** Cancer incidence before and after 20 years of age among 40 487 offspring of atomic-bomb survivors and controls

		**Age 1–19 years**		**Age 20+ years**		
**Cancer site**	**ICD-9^a^**	**Male**	**Female**	**Male**	**Female**	**Total**
*Solid tumors*	140–203, 225	15	9	234	317	575
Digestive	150–159	2	1	149	89	241
Respiratory	160–165	0	1	23	9	33
Female breast	174	0	0	0	88	88
Female genital organ	179–184	0	0	0	71	71
Male genital organ	185–187	1	0	14	0	15
Urinary	188–189	0	0	17	1	18
Brain and nervous	191,192,225	5	3	12	10	30
Thyroid	193	1	1	9	33	44
Other		6	3	10	16	35
						
*Hematopoietic tumors*	200–208	16	8	23	21	68
Leukemia	204–208	8	7	9	10	34
Lymphoma	200–202	8	1	14	11	34

a International Classification of Diseases, 9th Revision.

), 575 cases of solid tumors and 68 cases of hematopoietic tumors were diagnosed, representing a 1.4 and 0.2% cumulative incidence, respectively. Of these, 551 solid tumor and 44 hematopoietic tumor cases were diagnosed above age 20 years. For 3568 subjects with two exposed parents, 55 and 6 developed solid and hematopietic tumors, respectively. Age at onset ranged from 0.8 to 50.9 years with a median of 39.7, and attained age for cancer-free subjects ranged from 0.003 to 51.7 years with a median of 44.9. Mean length of follow-up was 39.3 years. As subjects aged, the number of solid tumors increased far more than that of hematopoietic tumors, particularly in the digestive system. Other major tumor sites were breast, genital organ, and thyroid for female subjects and respiratory system, prostate, and urinary system for male subjects. In the course of follow-up, 77% of the subjects had passed their 40th year.

Cancer incidence rates were no higher among the subjects with one or two parents exposed than among the reference subjects (Tables 3

**Table 3:** Adjusted risk ratio for solid tumor before and after 20 years of age in the offspring, according to parental preconception exposure to atomic bomb radiation

	**Age 1–19 years**				**Age 20+ years**			
**Dose (mSv)**	**No. of cases^a^**	**Risk ratio**	**95% CI**	***P*-value^b^**	**No. of cases^a^**	**Risk ratio**	**95% CI**	***P*-value^b^**
*Paternal exposure*								
0–4 (reference)	4	1.00		0.78	156	1.00		0.60
								
5–49	3	0.80	(0.17–3.68)		25	0.99	(0.63–1.49)	
50–149					23	0.89	(0.55–1.35)	
150–499					27	1.09	(0.71–1.63)	
500–4000					16	0.68	(0.39–1.10)	
Unknown					34	1.12	(0.76–1.61)	
								
Continuous dose (100 mSv)								
		1.03	(0.84–1.14)	0.66		0.96	(0.92–1.00)	0.07
								
*Maternal exposure*								
0–4 (reference)	11	1.00		0.97	256	1.00		0.99
								
5–49	11	0.98	(0.42–2.31)		43	0.89	(0.63–1.23)	
50–149					48	0.97	(0.70–1.31)	
150–499					49	1.02	(0.74–1.38)	
500–4000					34	1.02	(0.70–1.43)	
Unknown					43	0.99	(0.70–1.36)	
								
Continuous dose (100 mSv)		1.02	(0.88–1.12)	0.73		1.01	(0.98–1.04)	0.53

a In all, 55 offspring who developed solid cancer were conceived after both parents had been irradiated.

b Test for homogeneity of risk ratios among dose groups or test for trend of linear dose response; baseline rates adjusted for city, sex, year of birth, residency, and age at entry.

and 4

**Table 4:** Adjusted risk ratio for hematopoietic tumor before and after 20 years of age in the offspring, according to parental preconception exposure to radiation

	**Age 1–19 years**				**Age 20+ years**			
**Dose (mSv)**	**No. of cases^a^**	**Risk ratio**	**95% CI**	***P*-value^b^**	**No. of cases^a^**	**Risk ratio**	**95% CI**	***P*-value^b^**
*Paternal exposure*								
0–4 (reference)	7	1.00		0.47	11	1.00		0.86
								
5–49	7	1.07	(0.36–3.13)		3	1.68	(0.37–5.60)	
50–149					1	0.58	(0.03–3.06)	
150–499					2	1.10	(0.17–4.21)	
500–4000					1	0.63	(0.03–3.28)	
Unknown					0	Not estimated		
								
Continuous dose (100 mSv)		0.97	(0.73–1.09)	0.70		0.91	(0.65–1.07)	0.36
								
*Maternal exposure*								
0–4 (reference)	7	1.00		0.14	23	1.00		0.90
								
5–49	10	1.55	(0.59–4.28)		4	0.96	(0.28–2.53)	
50–149					3	0.66	(0.16–1.92)	
150–499					6	1.40	(0.51–3.24)	
500–4000					3	0.99	(0.23–2.87)	
Unknown					3	0.72	(0.17–2.10)	
								
Continuous dose (100 mSv)		1.04	(0.89–1.13)	0.58		0.97	(0.83–1.07)	0.63

a 6 offspring who developed hematopoietic tumor were conceived after both parents had been irradiated.

b Test for homogeneity of risk ratios among dose groups or test for trend of linear dose response; baseline rates adjusted for city, sex, year of birth, residency, and age at entry.

). For the subjects with two exposed parents, the adjusted risk ratio was 0.97 (95% Confidence Interval (CI) 0.70–1.34) for all cancers, 0.97 (95% CI 0.69–1.36) for solid tumors, and 0.95 (95% CI 0.32–2.64) for hematopoietic tumors. Cancer incidence rates were not positively associated with either paternal or maternal radiation exposure (*P*>0.1). Regarding a possible linear dose-response, the adjusted risk ratio of all cancers at 100 mSv was 0.96 (95% CI 0.92–1.00) for paternal exposure and 1.01 (95% CI 0.98–1.04) for maternal exposure. No trend toward increasing cancer incidence was found for increasing dose (*P*>0.05), but a decreasing incidence with increasing paternal dose was suggested (*P*=0.08). The adjusted risk did not change with age, calendar period, sex, or parental age at the time of the bombing (*P*>0.1). Findings were similar whether cancer occurred in childhood or young adulthood of the offspring.

## DISCUSSION

The present study is the first to examine cancer risks in early adulthood of offspring born to one or two atomic bomb survivors in Hiroshima and Nagasaki. Our results suggest that cancer incidence in children and young adults did not increase relative to parental exposure to atomic bombs. The estimates and 95% confidence intervals of risk ratios provide an indication of the magnitude of possible effects for all levels of preconception exposure. For example, the 95% upper confidence bound of risk ratios indicates that there might be a 34% increase in cancer incidence rates among the subjects with two exposed parents relative to the reference subjects. Our results are consistent with those obtained in the latest study of cancer mortality in childhood and young adulthood for the same cohort members (Izumi *et al*, 2003).

Our study is unique in certain respects. First, it involved a long-term follow-up of a large fixed cohort of a single ethnic group; second, the parents experienced single, whole-body exposures with a broad range of radiation dose and third, the reference group used for the present analysis, in contrast to earlier studies of offspring of survivors, included only subjects whose parents were in either city of Hiroshima or Nagasaki at the time of the bombing and whose radiation dose was 0–4 mSv. The aim of these criteria was to reduce the potential bias arising from unknown demographic factors that might affect baseline cancer incidence. The atomic-bomb survivor study has shown that dose-response analyses can be biased if based on a comparison group that is not comparable to the exposed subjects with respect to uncontrolled factors related to disease mortality (Cologne and Preston, 2001).

The present analysis is limited by a lack of detailed information on individual characteristics including socioeconomic, lifestyle, and biological factors that could affect cancer risks, particularly in early adult life. A mail survey of 24 000 offspring, which was started in 2001 and is to be completed in 2005, will provide information on education, alcohol consumption, smoking behavior, dietary habits, daily exercise, and morbidity. In 2002, RERF began a clinical programme for the survey participants that collects information on clinical and laboratory measurements; this will be incorporated in future analyses of these offspring.

A strength is that population-based tumor registry data allowed us to assess cancer risks more precisely than in previous cancer mortality studies. This, essentially, is because improved cancer therapy over the last few decades has decreased the fatality of some cancers so that a number of nonfatal cancer cases did not appear on death certificates. The proportion of cancer cases documented by death certificates only was 2.8%. Misclassification of cancer on death certificates may also have reduced the number of cancers. Comparisons of certified causes of death and autopsy diagnoses have shown that the percentage of cancer misclassification on death certificates (i.e. true cancers misclassified as noncancer) among atomic-bomb survivors was 10% for those aged under 65 years in the period 1961–1975 (Sposto *et al*, 1992). Histological confirmation of tumor cases from tissue registries has improved the quality of tumor registry data. Although tumors among the subjects who moved away from Hiroshima or Nagasaki were not included in the tumor registry data, the effects of migration on the baseline cancer rates were adjusted using the residence probabilities. The absence of cancer incidence data in the period 1946–1957 does not materially affect our results because the mortality data indicated that few offspring died from cancer during that period.

A careful interpretation is necessary for the suggested trend of decreasing adulthood cancer incidence for increasing paternal dose. Adjusted risk ratio of cancer mortality at 100 mSv was 0.95 (95% CI 0.87–1.01) for paternal exposure (Izumi *et al*, 2003), which is consistent with the results of the present analysis. The upper 95% confidence bound of adjusted risk ratio is slightly above 1.0 for both cancer incidence and mortality. Because residence probabilities were not affected by parental exposure to atomic bombs (unpublished data), migration may not be a plausible explanation for this finding. Thus, continuous follow-up data are necessary to examine this temporal pattern.

Risk assessment of cancer at advanced ages among the cohort members will require at least 30 more years of follow-up. In the present study, 77% of the offspring had passed their 40th year, but only 20% had reached 50 years of age. According to the National Life Table (Japanese Ministry of Health, Labour and Welfare), mean life expectancy (years) in Japan in 2001 was 78.07 for men and 84.93 for women. Such long life expectancies should make it possible to fully assess the effects of parental irradiation. Thus, further follow-up studies of the offspring of atomic-bomb survivors will help to address some of the limitations of our present analysis.

We concluded that we can not yet exclude an increase in cancer incidence among offspring of atomic bomb survivors, because of the small number of cases and the relatively young age of study subjects. However, our findings provide useful information of direct relevance to current public concerns about the health of offspring whose parents were exposed to ionizing radiation prior to conception, whether from therapy, their occupation, or their houses.
